# Cancer-related costs, the resulting financial impact and coping strategies among cancer survivors living in a setting with a pluralistic health system: a qualitative study

**DOI:** 10.3332/ecancer.2022.1449

**Published:** 2022-09-26

**Authors:** Noorulain FNU, Wai-Chee Kuan, Yek-Ching Kong, Ros Suzanna Bustamam, Li-Ping Wong, ShriDevi Subramaniam, Gwo-Fuang Ho, Hafizah Zaharah, Cheng-Har Yip, Nirmala Bhoo-Pathy

**Affiliations:** 1 Centre for Epidemiology and Evidence-Based Practice, Department of Social and Preventive Medicine, Faculty of Medicine, Universiti Malaya, 50603 Kuala Lumpur, Malaysia; 2 Department of Radiotherapy and Oncology, Kuala Lumpur Hospital, 50586 Kuala Lumpur, Malaysia; 3 Institute of Clinical Research, National Institutes of Health, 40170 Shah Alam, Malaysia; 4 Department of Clinical Oncology, Faculty of Medicine, Universiti Malaya, 50603 Kuala Lumpur, Malaysia; 5 Department of Radiotherapy and Oncology, National Cancer Institute, 62250 Putrajaya, Malaysia; 6 Subang Jaya Medical Centre, 47500 Subang Jaya, Malaysia

**Keywords:** cancer, cost, financial impact, coping strategies, unmet need, public-private healthcare

## Abstract

**Background:**

Evidence on the financial experiences of cancer survivors living in settings with pluralistic health systems remains limited. We explored the out-of-pocket costs, the resulting financial impact and the coping strategies adopted by cancer survivors in Malaysia, a middle-income country with a government-led tax-funded public health sector, and a predominantly for-profit private health sector.

**Methods:**

Data were derived from 20 focus group discussions that were conducted in five public and private Malaysian hospitals, which included 102 adults with breast, cervical, colorectal or prostate cancers. The discussions were segregated by type of healthcare setting and gender. Thematic analysis was performed.

**Results:**

Five major themes related to cancer costs emerged: 1) cancer therapies and imaging services, 2) supportive care, 3) complementary therapies, 4) non-medical costs and 5) loss of household income. Narratives on out-of-pocket medical costs varied not only by type of healthcare setting, clinical factors and socioeconomic backgrounds, but also by private health insurance ownership. Non-health costs (e.g. transportation, food) and loss of income were nonetheless recurring themes. Coping mechanisms that were raised included changing of cancer treatment decisions, continuing work despite ill health and seeking financial assistance from third parties. Unmet needs in coping with financial distress were especially glaring among the women.

**Conclusion:**

The long-term costs of cancer (medications, cancer surveillance, supportive care, complementary medicine) should not be overlooked even in settings where there is access to highly subsidised cancer care. In such settings, patients may also have unmet needs related to non-health costs of cancer and loss of income.

## Introduction

Cancer is a costly disease, not only for the health systems but also for patients. Compared to other chronic illnesses, it has been shown to be associated with higher out-of-pocket spending [1]. The term financial toxicity was coined to describe the devastating financial impact of cancer-related costs on affected households [2]. Financial toxicity associated with cancer care negatively impacts both cancer patients themselves and their families, who may be forced to make financial sacrifices to cope with cancer costs [3]. The COVID-19 pandemic has undeniably worsened the financial challenges faced by people living with cancer, particularly in the low- and middle-income countries (LMICs) [4, 5].

Malaysia is an upper middle-income country with a dual public-private healthcare system where patients are free to opt between public and private healthcare. Whereas the public hospitals in Malaysia are government-led and tax-funded, the private hospitals are predominantly run for profit and funded by out-of-pocket payments and private health insurance [6]. Country-specific analysis from the Association of Southeast Asian Nations (ASEAN) Costs in Oncology (ACTION) study had previously shown that 51% of cancer-stricken households in Malaysia experienced catastrophic expenditures within 1 year of cancer diagnosis, spending more than 30% of their combined household income for cancer-related expenses [7]. Among cancer patients seeking care in public hospitals, expenditure on non-medical goods and services was found to be an important driver of financial catastrophe, highlighting a lack of financial risk protection even in settings with universal health coverage [7].

Nonetheless, the ACTION study included a very small sample of cancer patients who were treated in the private sector. It was also limited by a lack of finer details on the specific types of costs that contributed to catastrophic payments [7], making it difficult to plan the needed resources and implement actionable programmes and policies to buffer the financial impact of cancer [8]. A recent qualitative inquiry had generated insightful evidence on the burden of direct and indirect costs following breast cancer as well as subsequent financial needs in a middle-income setting [9]. Nonetheless, additional research is needed to capture the perspectives of a wider patient population with different types of cancer who may also be at high risk for financial toxicity, apart from having specific unmet financial needs [10]. Furthermore, evidence on financial experiences of cancer patients who are treated in the private health sector is reprehensibly scarce in the LMICs.

We therefore undertook a qualitative inquiry to explore the lived financial experiences of patients with different types of cancers, who were treated in the public as well the private sectors in Malaysia. Specifically, we sought to understand the various types of cancer-related costs, the resulting financial impact and the coping strategies that were adopted by the cancer survivors.

## Methods

Data for the present study were obtained from a larger qualitative study conducted in Malaysia which examined the needs of cancer survivors in a setting with limited cancer supportive care services.

Participants were recruited between February 2018 and January 2019 from five centres located in Klang Valley, an urban conglomerate in Malaysia. The selected hospitals constituted a mix of public, academic and private facilities to ensure adequate representation of participants from diverse socio-demographic backgrounds. Eligible participants comprised men and women aged above 18 years old, who were diagnosed with either breast, cervical, colorectal or prostate cancer at least 3 months prior to the study. Patients with carcinoma *in situ* were excluded.

Eligible participants were identified and briefed about the study by their attending physicians during routine follow-ups. The research team contacted patients who expressed interest and invited them to join a focus group discussion (FGD). All FGDs were conducted at the respective hospitals where patients had their follow-ups. Each FGD comprised four to six participants and lasted about 2 hours. The FGDs, conducted in either English or Malay languages, were separated by gender to ensure that both the men and women with cancer were comfortable during the sessions and therefore were more open in discussing their challenges and needs. A focus group guide, which was tested and validated in a pilot study, was used by two moderators (YCK, LPW) during the FGDs. All FGDs were audio recorded and transcribed verbatim. Forward and backward translations were performed for FGDs conducted in the Malay language. Transcripts of the FGDs were supplemented by notes from the note taker. Written informed consent was obtained from all participants at the start of the FGDs. Participants were also asked to fill in a brief socio-demographic form. Data were collected until theoretical saturation, in which no new conceptual information emerged from subsequent FGDs.

The focus group guide included probes on the financial experiences of patients and their households. Specifically, probing questions comprised ‘what are the medical and non-medical costs incurred due to cancer’, ‘are the costs financially burdensome and why’, ‘how has cancer financially impacted you and your family’ and ‘how do you cope with the financial burden’.

Thematic analysis was performed using NVivo V12, whereby codes were extracted from the transcripts and analysed by two independent researchers to identify main themes and subthemes. Any discrepancies in data coding were resolved via triangulation and discussion.

## Results

Data saturation was reached after 20 FGDs; 12 FGDs with 64 women, 8 FGDs with 38 men. Majority of the participants were between 40 and 59 years old (49%), of Malay ethnicity (43%) and married (76%) (Table 1). About half hailed from low-income households, and two thirds of study participants were from public hospitals. Approximately 41% of patients had colorectal cancer, 30% with breast cancer, 20% with prostate cancer and 12% with cervical cancer. Median time since cancer diagnosis was 20 months (interquartile range: 11–38 months). Close to 60% of the cancer patients reported that they were diagnosed with advanced stages at initial diagnoses.

Five major themes of costs were identified, namely 1) cancer therapies and imaging services, 2) supportive care and medical aids/equipment, 3) complementary therapies, 4) non-medical costs and 5) loss of household income (Tables 2–6). Other important themes include coping strategies adopted by cancer patients as a result of cancer-related financial burden, and the ensuing unmet needs were discussed (Table 7).

### Costs of cancer

#### Cancer therapies and imaging services

When probed on the costs incurred from conventional cancer care, we found that the extent of financial burden was influenced by not only cancer site, healthcare setting and socioeconomic background but also by private health insurance status.

The high cost of chemotherapy was often brought up by those with colorectal and prostate cancer, while women with breast cancer lamented on the prohibitive costs of targeted therapies and certain hormone treatments. The need for long-term treatment was particularly emphasised by participants with breast cancer, whom had to continue paying for hormone therapies for a period of at least 5 years.

*‘I have to really monitor my finances because I know I will spend more for my medication, it’s a long-term thing…..I have to be on hormone therapy. It is not covered by my (private health) insurance. It is not cheap, one box costs about RM900 (US$225)’* (A 64-year-old female, stage 3 breast cancer, middle-income, private hospital, with private health insurance).

The need for long-term surveillance and imaging was also highlighted as a major source of financial burden by most participants.

*‘Besides treatment, I have other expenses. I have follow-ups, check-ups... I had to sell my jewelry to get money for my follow-ups’* (A 50-year-old female, stage 3 breast cancer, low-income, public hospital, no private health insurance).

*‘All the scans are actually very expensive. I spent over RM4000 (US$1000) just for scans’* (A 35-year-old female, stage 2 cervical cancer, middle income, public hospital, with private health insurance).

Among those with private health insurance coverage, limitation in the types of cancer treatments that were covered by some insurance policies and also underinsurance were cited as a source of financial burden irrespective of income status.

*‘My insurance can only cover surgery, it doesn’t cover for (adjuvant) treatment’* (A 74-year-old male, stage 2 colon cancer, low-income, academic hospital, with private health insurance).

Furthermore, it was also noted that patients were not clear on the type of private insurance policies that they owned, whereby even individuals hailing from high socioeconomic backgrounds were unable to distinguish between life insurance and private health insurance.

*‘I do have life insurance (not health insurance) but they don’t cover anything (cancer treatment). When it comes to insurance, everything is restricted for me. I really hope that the insurance agencies will give a chance to cancer patients’* (A 46-year-old female, stage 1 breast cancer, high income, private hospital, no private health insurance).

#### Supportive care and medical aids/equipment

Similar to that of cancer therapies, the financial burden of supportive care largely differed by cancer site. Nonetheless, the narrative that supportive care and medical aids/equipment were financially burdensome was similar across patients from the low-, middle- and high-income backgrounds. Study participants also explained that the medical aids/equipment that they needed were neither provided for free or near-free by the public healthcare system, nor reimbursed by private health insurance.

Among those with colorectal cancer, beside the costs of the colostomy bag itself, participants highlighted that they also had to pay for the base, glue, as well as special soap and powder for cleaning, all of which added up financially.

‘*There are 2 pieces, the bag and the base. It costs RM250 (US$65) just for the bag, not inclusive of the gum and the soap. So expensive’* (A 34-year-old female, stage 4 colorectal cancer, low income, public hospital, no private health insurance).

Although some social welfare organisations and religious societies in the country provided financial support to needy patients in purchasing colostomy bags, the study participants, while appreciative, highlighted issues with the quality of the subsidised bags such as fluid leakage and allergies. They also emphasised the relatively short-term nature of the financial assistance.

Among participants with breast cancer, many raised the burden of the high costs of breast prosthesis or supportive bras following mastectomy. Patients also highlighted the need to spend on physiotherapy, which although was medically necessary, added financial burden to their families. To cope with the costs, some women recounted foregoing them to minimise costs.

*‘It is expensive. When I think about buying the bra, I get scared. I was forced to spend RM250 (US$65) after my operation. It makes us so stressed’* (A 37-year-old female, stage 3 breast cancer, low income, public hospital, no private health insurance).

For participants with prostate cancer, their primary financial burden for supportive care was attributed to the need for medication for erectile dysfunction. As it is considered as a ‘lifestyle medication’, it is neither subsidised at the government hospitals nor covered by private health insurance. In many instances, patients reported paying out of pocket for it.

Irrespective of cancer site or stage, patients highlighted the financial burden arising from the need to purchase nutritional supplements that were prescribed to them such as formula milk.

#### Complementary therapies

Patients reported expenditures on traditional and complementary, which were deemed as substantial. Participants who were financially better off, particularly the women, further emphasised the need to spend on ‘high-quality’ food, and health supplements, all of which compounded their financial burden.

*‘I used a lot of my money... buy all the organic food, buy the quality and expensive things, so we really used a lot of money’* (A 49-year-old female, stage 2 breast cancer, high income, public hospital, with private health insurance).

#### Non-medical costs

Transportation and parking expenses were raised as important costs by many cancer patients, irrespective of income status or healthcare setting.

*‘Transport is a burden. I spent almost RM2000 (US$500) on ride hailing services. My husband doesn’t have time to fetch me and also because of the limited parking. So to and fro is already RM50 (US$13)’* (A 58-year-old female, colorectal cancer [stage unknown], high-income, private hospital, with private health insurance).

Participants also described increased household expenses such as costs for household help and caring of dependents.

*‘My mother-in-law used to live with us. It did become a problem when she was not well and I was getting into these treatments, especially chemo. That was a burden. That was why we hired a maid whose job was to take care of us’* (A 77-year-old male, prostate cancer stage unknown, high income, private hospital, with private health insurance).

#### Loss of household income

Irrespective of socioeconomic status, most participants described experiencing a drop in their household income. Study participants attributed the decline to not only the prolonged absence from work following cancer diagnoses, but also due to loss of productivity. It was sensed that female participants seemed more vulnerable to experience loss of household income, particularly those doing informal work.

*‘I am a babysitter. They (my customers) thought that because I am sick, I cannot care for their children and went on to find other babysitters...’* (A 60-year-old female, stage 2 breast cancer, low income, public academic hospital, no private health insurance).

Apart from their own employment issues, many also shared that the incomes of their household members were also affected, particularly their partners or caregivers. The reduction in household income was often described as an important consideration in making decisions on future healthcare seeking and follow-ups.

*‘During my chemo, my husband stopped working for three months. That added to my (financial) stress’* (A 48-year-old female, breast cancer stage unknown, low income, public academic hospital, no private health insurance).

### Coping strategies

#### Changing cancer treatment decisions

To cope with the financial burden of conventional and supportive care treatment, participants reported making decisions such as either foregoing or discontinuing recommended cancer therapies, opting for cheaper alternatives, consuming less than the recommended dose or transferring from private hospitals to public hospitals. One participant even recounted on how he chose to delay his cancer surgery after considering the costs of the colostomy bags.

*‘The medical fees [in private hospital] were terrible. I couldn’t afford it thus I got transferred to a public hospital’* (A 76-year-old male, stage 4 colon cancer, low-income, public academic hospital, no private health insurance).

#### Return to work

To reduce financial strain, some study participants described returning to work as soon as possible despite physical limitations and poor health status.

*‘I tried to fight because I was thinking about my five kids ... My husband’s income is only RM2000+(US$500) per month, so I am forced to (return to work)... I cannot just sit at home’* (A 34-year-old female, stage 4 colon cancer, low income, public hospital, no private health insurance).

Although many participants dire to resume normal working life following cancer diagnoses in order to cope with financial burden, workplace discrimination such as being demoted or laid off and ineligibility for promotion were brought up by a few female participants, which in turn affected their ability to cope with the substantial cancer costs.

*‘Upon returning to work after my cancer break, I have gone for promotion interviews three times. All three were rejected. The reason was, I am sick and I can’t give full commitment’* (A 41-year-old, female, stage 3 breast cancer, high income, public academic hospital, with private health insurance).

#### Seeking financial assistance

Participants in the study recounted that personal savings and financial support from third party helped in coping with the high financial burden of cancer. Third-party support included borrowing money from family and friends, and receiving financial payouts from private insurance policies, employment benefits, government support, religious bodies and non-profit associations.

*‘My son is working in the government, so it is all covered. At the same time, I have sufficient (private health) insurance coverage’* (A 62-year-old male, stage 3 prostate cancer, low income, public hospital, with private health insurance).

*‘Actually, I received my insurance (payout), so no problem now’* (A 65-year-old male, colorectal cancer stage unknown, middle income, public academic hospital, with private health insurance).

Nonetheless, most of the other participants in this study cited a lack of savings, absence of private health insurance or lack of access to workplace assistance or employer-sponsored health insurance. They also reported of not knowing where and how to seek formal financial assistance. Compared to male participants, the females, particularly those from the lower income backgrounds, more often described coping difficulties due to lack of support to obtain financial assistance or access social insurance schemes.

*‘I don’t have the government mandated savings for employees since I am not working (housewife).... some people asked me to apply for government welfare assistance for low-income groups but I am not sure how to apply for it’* (A 42-year-old female, stage 2 cervical cancer, low income, public hospital, no private health insurance).

*‘I feel that the (application) process to get help (financial assistance) is really hard. Like I don’t know where to go, how to get help’* (A 60-year-old female, stage 2 breast cancer, low income, public hospital, no private health insurance).

## Discussion

In this qualitative inquiry among patients living with cancer in a middle-income setting with a pluralistic health system, we gained insights into the numerous medical and non-medical cancer-related costs, and also the indirect costs attributed to income loss at the household level. Besides the active treatment phase, it was apparent that cancer-related expenditures were also incurred throughout the survivorship phase. While the role of private health insurance as an important source of financing in the private health sector was evident, underinsurance as well as lack of coverage for supportive care was concerning. Furthermore, study findings seem to hint that there may be gender disparities when it comes to coping with financial difficulties following cancer, where women appeared more vulnerable.

Our findings that the financial burden of cancer treatment costs was primarily driven by clinical factors are unsurprising, given that treatment modalities and frequencies, and their subsequent costs differ by cancer site and stage [11]. Notably, in our study, the costs for systemic cancer treatments were commonly brought up as financially burdensome due to the repeated payments needed for each treatment cycle, compared to one-off costs such as that for cancer surgery. This could also be attributed to the high costs of new innovator drugs, which are either not fully subsidised in public hospitals or not being included in the coverage, or exceeding the limit of coverage of private health insurance. Unfortunately, the price of new anticancer drugs is only expected to rise further; an analysis of 58 anticancer drugs approved by the Food and Drug Administration between 1995 and 2013 found that yearly launch prices on average increased by USD8500 [12]. In settings with universal health coverage, aside from challenges imposed by costly new cancer therapies, needy households may still struggle to cope with the cost of long-term standard treatment regimens even when only a minimal fee is imposed [9].

This study importantly highlights that the financial strain of cancer can extend well after the period of active treatment into the survivorship period. Expenditures for long-term therapies and cancer surveillance can be costly as the financial reserves of cancer-stricken households, such as insurance coverage, personal savings or household assets, may have already been depleted [13, 14]. Similar to previous study findings, expenditures on supportive care and medical aids, such as stoma bags, medication for erectile dysfunction and mastectomy bras, further exacerbate the financial strain faced by cancer patients as these are often long-term, and in certain circumstances, lifelong expenses [9, 15]. While much attention, and rightly so, has been given to reducing the costs of anticancer treatments, it is also crucial to address the long-term financial implications associated with daily living after active treatment. Follow-up care is crucial to monitor and detect cancer recurrence, while supportive care enables patients to thrive well beyond cancer. Our findings highlight an urgent need to also account for the financial burden associated with long-term cancer-related costs, including cancer surveillance tests, supportive care and complementary medicine in settings with universal health coverage.

Treatment decision-making, from seeking care after development of symptoms, to accepting and completing recommended surgeries and therapies, to adhering to routine follow-ups, can be influenced by the patients’ economic circumstances, including ownership of private health insurance, and existing assets and debts [10, 16]. While patients can dip into their financial reserves, such as savings and health insurance, these can be rapidly depleted due to the chronic nature of cancer. In households that do not even have enough savings to begin with, the financial implications of a cancer diagnosis can be especially devastating, leading to financial sacrifices and household impoverishment that affects every member of the household [3, 17]. The financial devastation is often exacerbated by the loss of income that is experienced by many cancer-stricken households [18]. Thus, many cancer patients will try to return to work as soon as they can to offset the financial pressure of their medical bills. Adequate paid medical leave and time off work for hospital follow-ups, workplace flexibility and anti-discriminatory workplace policies are vital to promote and facilitate employment retainment and return to work for people with cancer [19, 20].

While highly subsidised cancer care is available via public tertiary hospitals to all Malaysian citizens regardless of their insurance status, private health insurance remains as an important source of health financing in the country. Notably, ownership of private health insurance in Malaysia enables access to treatment in the private hospitals, where shorter waiting time to diagnostic services and cancer treatment, better access to innovator drugs and more advanced cancer surveillance compared to the public sector have traditionally been an attraction [21]. While the current study findings revealed that some patients deemed private health insurance as a lifesaver, unmet needs seem to exist as underinsurance appears to be a common theme apart from the inability to access supportive care. The Malaysia National Health Morbidity Survey 2019 reported that only 22% of the population owned private health insurance. As much as half of the Malaysian population, including about 71% of the poorest 20% of the whole nation do not have any means of supplementary financial coverage for medical treatment, other than the existing tax-funded health care coverage provided by the government [22]. Consistent with the study findings, there is an unmet need to design a robust national-level health insurance scheme or co-payment system to reimburse the rising cancer costs. While the public healthcare system is expanding and healthcare financing reforms are being discussed, policy makers will also need to plan ahead for the supplementary and complementary role of private health insurance in the country.

What was strikingly evident from this study is also that many Malaysians with cancer lacked basic knowledge even in terms of the type of private insurance (life insurance versus health insurance) or amount of coverage, let alone the reimbursement policies, or kinds of services that are not covered**.** This was irrespective of patients’ socioeconomic backgrounds. Hence, it is important to remember that to improve the status quo, any potential interventions aiming to improve health insurance literacy among the Malaysians need to be targeted to the young, healthy adults in Malaysia before they develop serious health-related sufferings.

In our study, despite both men and women experiencing comparable costs following cancer diagnoses, there is a hint that women were more vulnerable in terms of coping with the financial strain of cancer-related costs, including that of indirect costs. Previous studies have indeed reported a higher proportion of women who experienced income loss or employment disruption after a cancer diagnosis [23]. Even before their cancer diagnosis, women have been reported to be less likely to own adequate health insurance coverage, more likely to be unemployed, or employed in low-wage jobs or informal sectors, to have fewer saving and have poorer social support [24–27]. Coupled with pre-existing economic inequalities, it is conceivable that a diagnosis of cancer may render women more financially vulnerable due to their lack of access to economic resources. Nonetheless, we acknowledge that the male participants may have not fully disclosed the extent of their financial hardship due to cultural norms and societal expectations.

## Study strengths and limitations

To the best of authors’ knowledge, this is the first qualitative study exploring the financial experiences of patients with various cancers in a setting with dual-tiered system of healthcare. This allowed us to capture and understand the numerous financial needs experienced by our study participants who are also at various phases of the cancer continuum. Nonetheless, as we only included those with either breast, cervical, prostate or colorectal cancers, our study findings may not necessarily be generalisable to patients with other cancers, who may have additional financial needs. Furthermore, study participants were recruited from hospitals in Klang Valley, Malaysia, which is an urban area. However, the study sites are major oncology referral centres in the country, which also caters to participants hailing from other sub-urban/rural states. Future studies should be conducted to validate our findings among patients with other cancers, as well as those receiving cancer treatments in sub-urban or rural areas.

## Conclusion

In settings with universal health coverage, it is also important to not neglect the financial burden associated with long-term costs of cancer, particularly for long-term medications, cancer surveillance tests, supportive care and also complementary medicine. Besides highlighting the need to improve ‘health insurance literacy’ among the general population, this study underscores the urgent need for policy-makers in the LMICs to confront the role that private health insurance will play in their health systems and regulate the sector appropriately to ensure that it serves public goals of universal coverage and equity [28]. On the same note, the indirect costs of cancer such as delayed return to work, decreased productivity at workplace and costs of informal caregiving must be recognised and addressed by the governments in the LMICs, as it is clearly beyond the scope of healthcare systems per se. Lastly, the current study provides insight that financial assistance programmes need not only be sensitive to the clinical characteristics and socioeconomic backgrounds of patients, but also to gender. To this end, it is felt that a feminist economic agenda is needed when planning cancer care in our setting, as women (and their families) may benefit from targeted support to cope with the financial strain of cancer. On a wider note, a financial navigation programme connecting patients to the appropriate financial resources is urgently needed for people dealing with cancer, also in settings with universal health coverage.

## Statements and declarations

### Funding

This study was supported through an unrestricted educational grant from PhAMA (Pharmaceutical Association of Malaysia) provided to Nirmala Bhoo-Pathy through her institution (Universiti Malaya). The funders had no role in the design of the study, data collection and analysis, preparation of the manuscript or decision to publish.

### Conflicts of interest

The authors have no relevant financial or non-financial interests to disclose.

### Authors’ contributions

Yek-Ching Kong and Nirmala Bhoo-Pathy contributed to the study conception and design. Material preparation and data collection were done by Yek-Ching Kong, Ros Suzanna Bustamam, ShriDevi Subramaniam, Gwo-Fuang Ho, Hafizah Zaharah, Cheng-Har Yip and Li-Ping Wong. Data analysis was performed by Noorulain FNU, Yek-Ching Kong, Wai-Chee Kuan and Nirmala Bhoo-Pathy. The first draft of the manuscript was written by Noorulain FNU, Yek-Ching Kong, Wai-Chee Kuan and Nirmala Bhoo-Pathy. All authors commented on previous versions of the manuscript. All authors read and approved the final manuscript. Study supervision and review done by Nirmala Bhoo-Pathy.

### Ethics approval

The study received ethical approval from the Medical Research and Ethics Committee [NMRR-17-3361-39122], University Malaya Medical Centre Medical Ethics Committee [201831-6061] and Subang Jaya Medical Centre [201809.2].

### Consent to participate

Written informed consent was obtained from all participants at the start of the FGDs.

## Figures and Tables

**Table 1.** Demographic characteristics of study participants.

	Overall (*N* = 102) *n* (%)	Female (*n* = 64) *n* (%)	Male (*n* = 38) *n* (%)
Age, years			
<40	19 (18.6%)	15 (23.4%)	4 (10.5%)
40–59	50 (49.0%)	40 (62.5%)	10 (26.3%)
60 and above	33 (32.4%)	9 (14.1%)	24 (63.2%)
Ethnicity			
Malay	44 (43.1%)	36 (56.3%)	8 (21.2%)
Chinese	37 (36.3%)	16 (25.0%)	21 (55.3%)
Indian	17 (16.7%)	11 (17.2%)	6 (15.8%)
Others	4 (3.9%)	1 (1.6%)	3 (7.9%)
Monthly income status			
Low (≤MYR3,000)	38 (38.0%)	25 (39.1%)	13 (36.1%)
Middle (MYR3,001–RM6,000)	31 (31.0%)	20 (31.3%)	11 (30.6%)
High (>MYR6,000)	31 (31.0%)	19 (29.7%)	12 (33.3%)
Missing	2	0	2
Type of hospital			
Public (Ministry of Health)	47 (55.9%)	37 (57.8%)	20 (52.6%)
Public (University)	25 (24.5%)	16 (25.0%)	9 (23.7%)
Private	20 (19.6%)	11 (17.2%)	9 (23.7%)
Cancer site			
Colorectum	42 (41.2%)	22 (34.4%)	20 (52.6%)
Breast	30 (29.4%)	30 (46.9%)	-
Cervix	12 (11.8%)	12 (18.8%)	-
Prostate	18 (17.6%)	-	18 (47.4%)

**Table 2.** Subthemes for cancer therapies and imaging services.

Subthemes	Representative quotes
Cancer medications (short-term and long-term)	*‘I have to really monitor my finances because I know I will spend more for my medication, it’s a long-term thing….I have to be on hormone therapy. It is not covered by my insurance. It is not cheap, one box costs about RM 900(US$225)’* (A 64-year-old female, stage 3 breast cancer, middle-income, private hospital, with private health insurance) *‘Besides treatment, I have other expenses. I have follow-ups, check-ups... I had to sell my jewelry to get money for my follow-ups’* (A 50-year-old female, stage 3 breast cancer, low-income, public hospital, no private health insurance) *‘My insurance can only cover for surgery, it doesn’t cover for (other) treatment’* (A 74-year-old male, stage 2 colorectal cancer, low-income, academic hospital, with private health insurance) *‘I do have life insurance policy (but not private health insurance) but they don’t cover anything (cancer treatment). When it comes to insurance, everything is restricted for me. I really hope that the insurance agencies will give a chance to cancer patients’* (A 46-year-old female, stage 1 breast cancer, high-income, private hospital, no private health insurance)
Cancer imaging	*‘I make sure every year I go for my PET scan, even if I have to pay from my own pocket, I will still do it… I want to know whether it has grown and went somewhere else’* (A 52-year-old female, stage 4 breast cancer, high income, private hospital, with private health insurance) *‘All the scans are actually very expensive. I spent over RM4000 just for scans’* (A 35-year-old female, stage 2 cervical cancer, middle income, public hospital, with private health insurance)
Cancer surveillance	*‘Besides treatment, I have other expenses. I have follow-ups, check-ups... I had to sell my jewelry to get money for my follow-ups’* (A 50-year-old female, stage 3 breast cancer, low-income, public hospital, no private health insurance)

**Table 3.** Subthemes for supportive care.

Subthemes	Representative quotes
Medical aids/equipment	‘*There are 2 pieces (colostomy bag), the bag and the base. It costs RM250 (US$63) just for the bag, not inclusive of the gum and the soap. So expensive’* (A 34-year-old female, stage 4 colorectal cancer, low-income, public hospital, no private health insurance) *‘Every month I have to pay hundreds. It (one colostomy bag) can only last for 2-3 days… we have to change the bag frequently. The expenses every month just for this is really high. No subsidy. I tried applying but it is so hard to get’* (A 38-year-old female, colorectal cancer stage unknown, low-income, public hospital, no private health insurance) *‘One non-governmental organization gave it (colostomy bag) for free for 6 months. But I can’t use it because I am allergic to it.... Let’s say I have to use the stoma bag permanently for my whole life, I can’t get the bag for my entire life. That’s what the social welfare department (from the hospital) told me’* (A 34-year-old female, stage 4 colorectal cancer, low-income, public hospital, no private health insurance) *‘It is expensive. When I think about buying the bra, I get scared. I was forced to spend RM250(US$63) after my operation. It makes us so stressed’* (A 37-year-old female, stage 3 breast cancer, low-income, public hospital, no private health insurance)
Management of side effects of cancer therapy – impotence	*‘It (medication for erectile dysfunction) is not essential medicine, it is lifestyle medication. So they don’t give. Even insurance companies don’t pay for lifestyle medicine’* (A 58-year-old male, prostate cancer stage unknown, high-income, private hospital, with private health insurance)
Food supplements	*‘The milk is really expensive you know, RM40+ (USD10) for one small tin. And you have to take a lot of scoops, 6 scoops!’* (A 34-year-old Malay female, stage 4 colorectal cancer, low-income, public hospital, no private health insurance)
Physiotherapy	*‘That also depends because of the cost. If they suggest 3 (physiotherapy) sessions, I just go for one to save cost’* (A 48-year-old female, breast cancer stage unknown, middle-income, public academic hospital, no private health insurance)

**Table 4.** Subtheme for complementary therapies.

Subtheme	*Representative quotes*
Complementary therapies	*‘I used a lot of my money... buy all the organic food, buy the quality and expensive things, so we really used a lot of money’* (A 49-year-old female, stage 2 breast cancer, high-income, public hospital, with private health insurance)

**Table 5.** Subthemes for non-medical costs.

Subthemes	*Representative quotes*
Transportation	*‘Transport is a burden. I spent almost RM2000 (US$500) on ride hailing services. My husband doesn’t have time to fetch me and also because of the limited parking. So to and fro is already RM50 (US$13)’* (A 58-year-old female, colorectal cancer stage unknown, high-income, private hospital, with private health insurance)
Parking costs	*‘The rate for parking for first 1 hour is RM 1(25cents) and subsequent hours RM 2(50 cents). One day I have to pay more than RM 10 (US$2.5)’* (A 63-year-old male, colorectal cancer stage unknown, middle-income, public academic hospital, with private health insurance)
Household help	*‘My mother-in-law used to live with us. It did become a problem when she was not well and I was getting into these treatments, especially chemo. That was a burden. That was why we hired a maid whose job was to take care of us’* (A 77-year-old male, prostate cancer stage unknown, high-income, private hospital, with private health insurance)

**Table 6.** Subthemes for loss of household income.

Subthemes	*Representative quotes*
Loss of productivity	*‘I had total loss of earnings. Every day I have to go to the toilet for 8 times... How am I supposed to lecture?’* (A 51-year-old male, colorectal cancer stage 3, middle-income, public hospital, no private health insurance) *‘I am running my own business. It certainly has impacted my income since I am my own boss… Incontinence is quite a problem. I had to hide it (colostomy bag). I couldn’t go to court (workplace)...’* (A 49 year-old male, colon cancer, married, high-income, private hospital, with private health insurance)
Unemployment and difficulties at work place	*‘I am a babysitter. They (my customers) thought that because I am sick, I cannot care for their children and went on to find other babysitters’* (A 60-year-old female, breast cancer stage 2, low-income, public academic hospital, no private health insurance) *‘I was forced to resign… no job, forced resignation, no income. I was admitted in April, she (employer) withheld my salary for April and May. They didn’t pay my salary, I was so worried on how to pay my hospital bills’* (A 55-year-old-female, stage 2 cervical cancer, public hospital, high-income, no private health insurance) *‘Upon returning to work after my cancer break, I have gone for promotion interviews three times. All three were rejected. The reason was, I am sick and I can’t give full commitment’* (A 41-year-old, female, breast cancer stage 3, high-income, public academic hospital, with private health insurance)
Impact on employment of caregiver	*‘During my chemo, my husband stopped working for three months. That added to my (financial) stress’* (A 48-year-old female, breast cancer stage unknown, low-income, public academic hospital, no private health insurance)

**Table 7.** Subthemes for coping strategies.

Subthemes	*Representative quotes*
Changing cancer treatment decisions	*‘My savings was enough for 3 cycles of chemo only. I have to stop the treatment due to financial issue. My doctor suggested taking a targeted medication but it is expensive. Even if the social welfare covers for half of the cost, I would still need to pay RM 2,000 (US$500) for 2 weeks. I could not afford it… I had to put on hold the PET scan because it was expensive’* (A 26-year-old male, stage 4 colon cancer stage, middle-income, public academic hospital, no private health insurance) *‘One of the doctors recommended me to drink (special) milk because I don’t have appetite to eat... If I take 6 scoops, it will finish really fast, so I just use 3 scoops’* (A 42-year-old female, cervical cancer stage 2, low-income, public hospital, no private health insurance) *‘The medical fees [in private hospital] were terrible. I couldn’t afford it thus I got transferred to a public hospital’* (A 76-year-old male, stage 4 colon cancer, low-income, public academic hospital, no private health insurance) *‘That’s why I delayed my surgery in the first place, I cannot afford to buy the stoma bag’* (A 46-year-old male, stage 3 colorectal cancer, low-income, public hospital, no private health insurance)
Early return to work	*‘I was thinking about my five kids ... My husband’s income is only RM2000+(US$500) per month, so I am forced to (return to work)... I cannot just sit at home’* (A 34-year-old female, stage 4 colon cancer, low-income, public hospital, no private health insurance) *‘I was working after my diagnosis. I did not apply for cancer leave. I only took off during my treatment... It was actually a big burden for me, because I desperately needed rest. I was given a light duty letter by the doctor, but at my department it didn’t happen’* (A 40-year-old female, stage 4 breast cancer, high-income, public academic hospital, with private health insurance)
Seeking financial assistance	*‘My son is working in the government, so it (treatment) is all covered. At the same time, I have sufficient insurance coverage’* (A 62-year-old male, stage 3 prostate cancer, low-income, public hospital, with private health insurance) *‘At that time, I was fortunate that it (cancer surgery, chemotherapy) was covered by company (employer-sponsored) insurance, so not much financial problems’* (A 46-year-old female, stage 1 breast cancer, high-income, private hospital, with private health insurance) *‘Actually, I received my insurance (payout), so no problem now’* (A 65-year-old male, colorectal cancer stage unknown, middle-income, public academic hospital, with private health insurance) *‘I don’t have the government mandated savings for employees since I am not working (housewife).... some people asked me to apply for government welfare assistance for low-income groups but I am not sure how to apply for it’* (A 42-year-old female, stage 2 cervical cancer, low income, public hospital, no private health insurance) *‘I feel that the (application) process to get help (financial assistance) is really hard. Like I don’t know where to go, how to get help’* (A 60-year-old female, stage 2 breast cancer, low income, public hospital, no private health insurance)
